# Consistency enhancement of model prediction on document-level named entity recognition

**DOI:** 10.1093/bioinformatics/btad361

**Published:** 2023-06-01

**Authors:** Minbyul Jeong, Jaewoo Kang

**Affiliations:** Department of Computer Science and Engineering, Korea University, Seoul 02841, Republic of Korea; Department of Computer Science and Engineering, Korea University, Seoul 02841, Republic of Korea; Interdisciplinary Graduate Program in Bioinformatics, Korea University, Seoul, Republic of Korea; AIGEN Sciences, Seoul 04778, Republic of Korea

## Abstract

**Summary:**

Biomedical named entity recognition (NER) plays a crucial role in extracting information from documents in biomedical applications. However, many of these applications require NER models to operate at a document level, rather than just a sentence level. This presents a challenge, as the extension from a sentence model to a document model is not always straightforward. Despite the existence of document NER models that are able to make consistent predictions, they still fall short of meeting the expectations of researchers and practitioners in the field. To address this issue, we have undertaken an investigation into the underlying causes of inconsistent predictions. Our research has led us to believe that the use of adjectives and prepositions within entities may be contributing to low label consistency. In this article, we present our method, *ConNER*, to enhance a label consistency of modifiers such as adjectives and prepositions. By refining the labels of these modifiers, ConNER is able to improve representations of biomedical entities. The effectiveness of our method is demonstrated on four popular biomedical NER datasets. On three datasets, we achieve a higher F1 score than the previous state-of-the-art model. Our method shows its efficacy on two datasets, resulting in 7.5%–8.6% absolute improvements in the F1 score. Our findings suggest that our ConNER method is effective on datasets with intrinsically low label consistency. Through qualitative analysis, we demonstrate how our approach helps the NER model generate more consistent predictions.

**Availability and implementation:**

Our code and resources are available at https://github.com/dmis-lab/ConNER/.

## 1 Introduction

Named entity recognition (NER) is a task that plays a vital role in determining entity boundaries and classifying categories of named entities. NER is a fundamental part of biomedical applications, which typically utilize sentence-level models, but require the models to evaluate at a document level (Kim *et al.* 2019, Wei *et al.* 2019, Lee *et al.* 2020, Lewis *et al.* 2021, Weber *et al.* 2021, Sung *et al.* 2022). The shift from sentence-level models to document-level models seems inevitable. This presents a challenge that extension from a sentence model to a document model is not always straightforward. Recent studies in both the general and biomedical domains have attempted to train and evaluate NER models in a document-level context, with promising results (Wei *et al.* 2019, Yamada *et al.* 2020, Yu *et al.* 2020, Gui *et al.* 2021, Wang *et al.* 2021, Weber *et al.* 2021).

There are several advantages to using document-level NER models in biomedical applications. These models provide a better way to bridge the gap between research and application fields by considering the document as a whole. While previous studies (Cho and Lee 2019, Perera *et al.* 2020, Jeong and Kang 2021) have leveraged sentence-level NER models, these models are not well-suited for applications that require an evaluation of the document as a whole. Document NER models provide accurate and consistent predictions due to the completeness of the context they consider. Recent works (Yamada *et al.* 2020, Gui *et al.* 2021, Wang *et al.* 2021) have shown that using document-level contexts improves the accuracy of the models. Based on these findings, our goal is to train the model to make accurate and consistent predictions within the same document.

**Figure 1:**
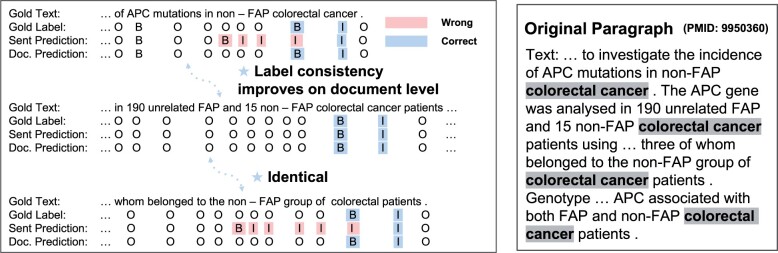
Overview of motivating examples. (Left) We provide an example with a gold label, sentence model prediction, and document model prediction. Many biomedical entities contain adjectives or prepositions within them. By providing sufficient context, we aim to improve the prediction of label consistency for those adjectives, such as “colorectal.” (Right) It is worth noting that the majority of biomedical datasets consist of a golden paragraph such as a combination of the title and abstract of biomedical literature (PubMed).

However, we ideally hope that all predictions are accurate, but since that is not always the case in reality, we aim to create NER models that make consistent predictions despite inaccuracies. The need for consistent predictions in the models is crucial for many biomedical applications, as inconsistent predictions can lead to significant errors in information extraction and knowledge discovery. We provide our motivating example in Fig. 1, where the mention “colorectal cancer” is an entity of the disease type. However, predicting such a mention is challenging in a sentence context due to the incompleteness of the context. As a result, the sentence-based NER model may produce errors in predicting “non—FAP” or “colorectal.” This is where document-level NER models come in, as they provide sufficient label consistency, which refers to the degree of label assignment across the dataset, to learn representations of “colorectal” by taking into account the context of the entire document. In this way, document-level NER models are able to make more consistent predictions than sentence-level models.

**Figure 2:**
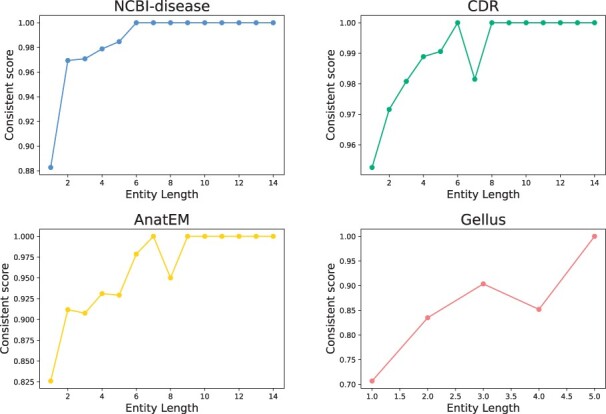
Consistent score per entity length. The *x*-axis denotes the entity length ($\phi_{\mathrm{eLen}}$), and the *y*-axis denotes the consistent score ($\phi_{\mathrm{eCon}}$). Short lengths of entities show low consistent scores due to the low label consistency of the modifier tokens.

Although document-level NER models show consistent predictions and are much better than sentence-level models, they still fall behind our expectations (Table 1). In the NCBI-disease dataset (Doğan *et al.* 2014), models trained on document contexts continue to produce 64% errors on predicting biomedical entities that contain modifiers (i.e. adjectives and prepositions), such as “*primary, hereditary*, and *congenital.*” (We suggest the breakdown error cases in the Supplementary Data.) This is because modifiers are used as both entity and nonentity tokens depending on the context situation, making them difficult to predict (Table 2). Furthermore, the modifiers that frequently occur at short entity lengths exhibit a low label consistency (Fig. 2).

**Table 1.** Results on four biomedical NER benchmarks.^a^

Context	Model	Evaluation (F1)				
		NCBI-disease	CDR	AnatEM	Gellus	Avg.
Sentence	BioBERT	89.0	89.1	73.9	54.9	76.7
	BioLM	88.3	89.5	74.9	55.9	77.2
Document	BioBERT	89.1	89.9	77.1	56.3	78.1
	BioLM	88.5	90.2	80.3	56.9	79.0

a We use BioBERT (Lee *et al.* 2020b) and BioLM (Lewis *et al.* 2020) to compare the predictions between different context levels. We report the F1 score to evaluate the models.

**Table 2.** Case studies of label consistency in the modifier tokens.^a^

Data	Criteria	Modifier token examples					
		Primary	Genetic	Hereditary	Inherited	Congenital	Abnormal
Train	$\phi_{\text{tCon}}$	0.11	0.79	0.13	0.58	0.27	0.94
Dev	$\phi_{\text{tCon}}$	0.13	0.69	0.13	0.51	0.27	0.97

a The case was taken from the NCBI-disease dataset (Doğan *et al.* 2014). $\phi_{\text{tCon}}$ refers to the token label consistency. Modifier tokens pose a challenge for NER models as they exhibit a low consistency score, making their prediction difficult.

To address the aforementioned errors, we present *ConNER*, a novel method that enhances the label consistency of modifier prediction. The process begins by feeding an abstract of biomedical literature into a biomedical pretrained language model, such as BioBERT (Lee *et al.* 2020b) or BioLM (Lewis *et al.* 2020), to generate context-dependent representation (Section 3.1). On top of this pretrained model, we introduce label refinement to make a consistent label distribution [The label distribution refers to the probability distribution of BIO tagging scheme (Ramshaw and Marcus, 1999).] of uncertain tokens within entities (Section 3.2). Our loss term for biomedical entities is designed to resemble the label distribution on two different architectures: fully connected layers (MLP) and bidirectional long-short term memory (BiLSTM) architecture. The MLP is used as the main classification layer to generate draft labels of the raw text, while the BiLSTM architecture is used to generate label distributions of biomedical entities, which can refine the draft labels with enhanced label consistency of corresponding modifiers within entities. To evaluate the effectiveness of the proposed ConNER approach, we use four biomedical benchmarks. On three datasets, we achieve a higher F1 score than previous state-of-the-art models (Lewis *et al.* 2020, Wang *et al.* 2021). Our model demonstrates a higher label consistency and proves its efficacy on the AnatEM (Pyysalo and Ananiadou 2014) and Gellus (Kaewphan *et al.* 2016) datasets, with 7.5%–8.6% absolute improvements in F1 score, as shown in Section 4.3. We provide an interpretation of the effectiveness of our method for a dataset that intrinsically has a low label consistency score (Section 5.4).

The main contributions of this study are: (i) we examine the reasons behind inconsistent predictions made by document NER models in biomedical domains and find that modifiers such as adjectives and prepositions have a low label consistency score and are a source of errors. (ii) We introduce a new method, *ConNER*, which improves the label consistency of modifier prediction. (iii) Through experimental results, we demonstrate that ConNER significantly enhances the accuracy of document NER models, achieving the highest level of label consistency. (iv) For different tasks related to low label consistency, we show that ConNER outperforms various baselines, and we analyze the factors influencing its performance.

## 2 Background

### 2.1 Named entity recognition

The goal of Named Entity Recognition (NER) is to identify and classify specific instances, such as a disease, chemical, species, or any other miscellaneous, within a corpus of text. This task involves extracting and categorizing named entities using predefined entity tags. One commonly used tagging scheme is BIO tagging (Ramshaw and Marcus 1999), where a “B” prefix indicates the beginning of an entity, an “I” prefix indicates being inside of an entity, and an “O” prefix indicates not belonging to an entity. The NER task can be approached using two different decoding strategies: tag-independent decoding [such as using a MLP (Collobert *et al.* 2011)] and tag-dependent decoding [such as using a Conditional Random Field (Lafferty *et al.* 2001)]. In this study, we only utilize the tag-independent decoding strategy.

### 2.2 Attribute definition

Following previous works (Fu *et al.* 2020, 2021), we use the term *attribute* as a value that characterizes the properties of an entity that may be correlated with performance. The authors introduced attributes bridging the gap between the final performance (we use the F1 score) and interpretable evaluation based on model predictions. Assuming that one attribute is given, the evaluation set of NER tasks naturally partitions into several interpretable buckets, we investigate whether the attribute affects the final performance bucket-wise.

**Figure 3:**
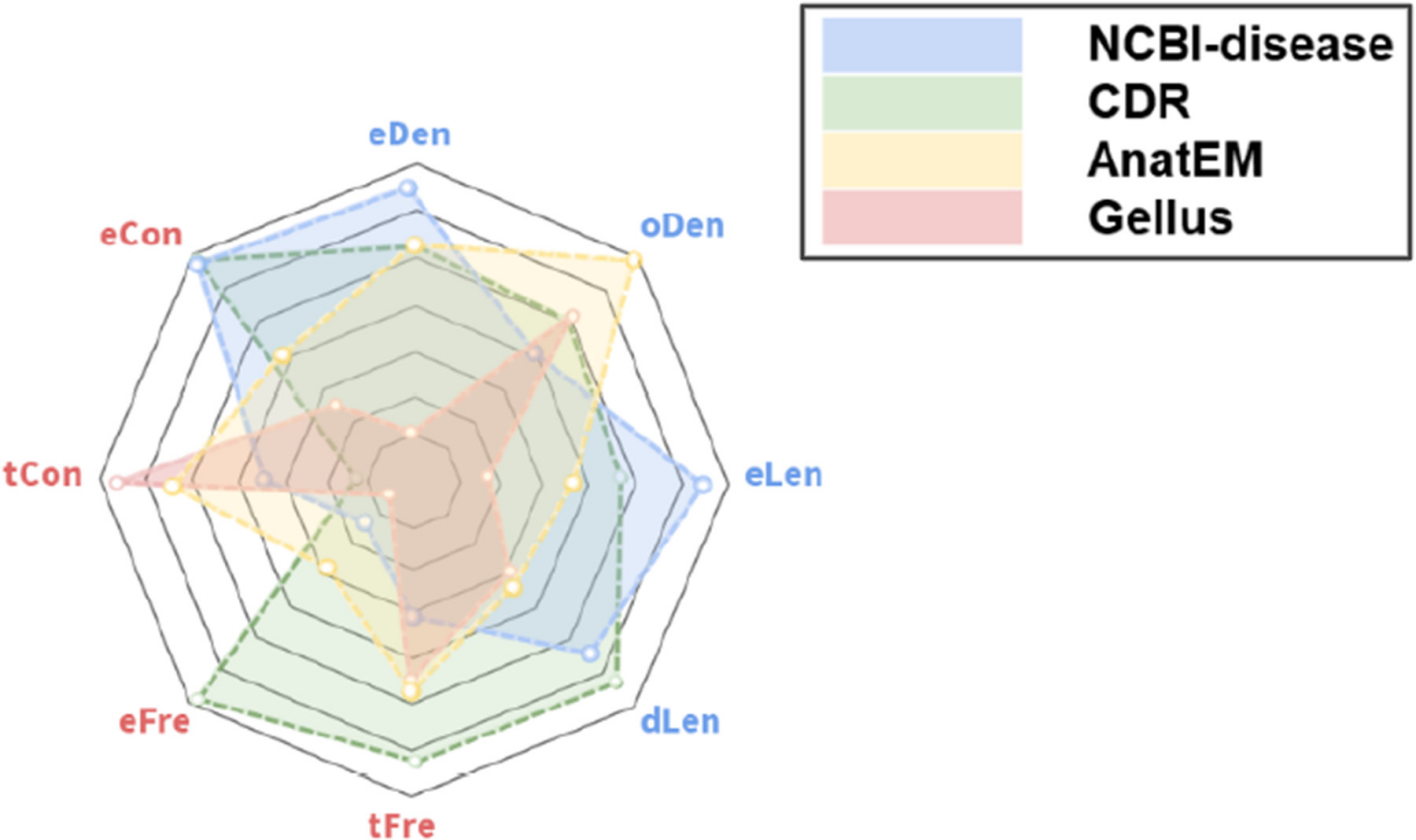
Overview of dataset biases. Each dot denotes an attribute score of entities. Intuitively, these intrinsic differences in datasets explain what factors have a significant influence on improving performance.

Formally, we define notations to facilitate the definition of attributes. Given a set of documents *D*, entity tagging aims to extract a set of entities E as tokens. (We denote a token as a sequence of characters that typically represent a word from the input text. This is encoded as a numerical vector used as inputs to the pretrained model.) We first denote an entity set E as an argument. Specifically, (Fu *et al.* 2020) introduced a feature function ϕ(⋅) to aggregate features to interpret the properties of tagged entities in an entity set E: where *x* and **x** denotes a token and an entity respectively, and $\phi_{\text{eLen}}$ refers to the length of an entities, and $\phi_{\text{label}}(\cdot )$ denote a label of the corresponding argument, and $\phi_{\text{tCon}}(x)$ and $\phi_{\text{eCon}}(x)$ refers to measuring how consistently a certain token and entity is assigned to a predefined label, respectively. For example, in the NCBI-disease dataset (Doğan *et al.* 2014), a “*primary*” token appears as an entity with an 11% probability ($\phi_{\text{tCon}}=0.11$) and a “*colorectal cancer*” entity consists of two tokens (“*colorectal*,” and “*cancer*”) resulting in a length of 2. In addition, there are various attribute functions such as frequency ($\phi_{\text{eFre}}$, $\phi_{\text{tFre}}$), context length ($\phi_{\text{dLen}}$), the density of out-of-vocabulary ($\phi_{\text{oDen}}$), the density of entities ($\phi_{\text{eDen}}$), etc. But in this case, we only use the above three. We use attribute functions to interpret which dataset attributes have an impact on performance improvement (Fig. 3).


(1) $$\phi_{\text{eLen}}(x)=|x|$$

(2) $$\phi_{\text{tCon}}(x)=\frac{\left|\{\varepsilon |\phi_{\text{label}}(\varepsilon )=\phi_{\text{label}}(x),\forall \varepsilon \in D(E)\}\right|}{\left|D(E)\right|},$$

(3) $$\phi_{\text{eCon}}(x)=\frac{\left|\{\varepsilon |\phi_{\text{label}}(\varepsilon )=\phi_{\text{label}}(x),\forall \varepsilon \in D(E)\}\right|}{\left|D(E)\right|},$$

## 3 Materials and methods

**Figure 4:**
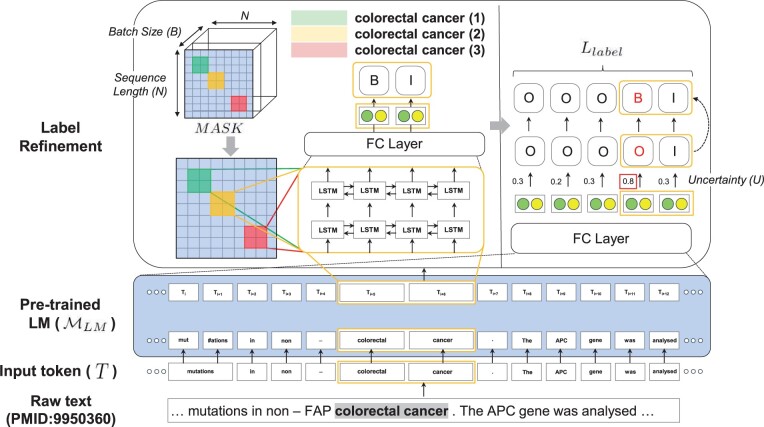
Overview of ConNER performing label refinement on biomedical entities. The abstract of biomedical literature is fed into the pretrained language model $M_{\text{LM}}$ to produce context-dependent representation. In the label refinement process, we use a mask tensor *MASK* to locate the positions of biomedical entities and feed it into the BiLSTM architecture $M_{\text{LSTM}}$ to enhance the label distribution of modifiers within entities that cause low label consistency. A fully connected layer is used to calculate an uncertainty score *U*, which determines which tokens need to be adjusted for their predicted label.

In this section, we embark on learning the document-level model for the named entity recognition (NER) task. Our objective is to enhance the label distributions of biomedical entities by improving their label consistency (Section 3.1). We present our label refinement process for biomedical entities, which helps in refining the label distribution of uncertain tokens within entities (Section 3.2). Finally, we explain how we use a loss term to make the label distribution of biomedical entities resemble the label distribution on two different architectures. The overall structure of ConNER approach is illustrated in Fig. 4.

### 3.1 Document named entity recognition

ConNER consists of a pretrained language model $M_{LM}$ and label-refinement process, as shown in Fig. 4. For document tagging, we use abstracts of biomedical literature as raw input and make predictions for each word. Let $D_{i}=\{T_{i1},T_{i2},\ldots ,T_{iN}\}$ represent a sequence of tokens, where *N* denotes the total number of tokens in a document $D_{i}$. First, we apply a biomedical pretrained language model [e.g. BioLM (Lewis *et al.* 2020)] to obtain context-dependent representations for each token: $T_{i1},T_{i2},\ldots ,T_{iN}\in R^{d}$, as follows, where *h* denotes the context-dependent representation of raw input, and *P* denotes the probability distribution of a main classification layer, and *d* denotes the final hidden dimension of language model $M_{\text{LM}}$, and *C* denotes the number of predefined entity types per dataset. We use tag-independent decoding (MLP) on the model $M_{\text{LM}}$ as a main classification layer, which is useful for predicting long-named entities (Fu *et al.* 2020, Jeong and Kang 2021). Formally, we can define our classification loss $L_{\text{class}}$ using cross-entropy objectives to optimize our model as follows: where $y_{j}$ denotes the ground-truth label and $p_{j}$ denotes the probability of the main classification layer.


(4) $$h=M_{LM}([T_{i1}:T_{iN}])\in R^{N\cdot d}$$

(5) $$p=MLP(h)\in R^{N\cdot C}$$

(6) $$L_{\text{class}}=-\frac{1}{N}\sum_{j=1}^{N}y_{j}\cdot \;\text{log}(p_{j})$$

### 3.2 Label refinement on biomedical entities

Overall, we present a label refinement process aimed at improving the label distributions of uncertain tokens within biomedical entities. Our approach considers the entity-length attribute $\phi_{\text{eLen}}$ to achieve this goal. To encourage label distribution, we add a loss term in two different models: the main classification layer and a BiLSTM architecture (Lafferty *et al.* 2001). The MLP layer is used to produce the label distribution, along with the uncertainty score and draft label for each token. The draft label refers to the classified label which derives from a max probability of label distribution. The BiLSTM architecture, on the other hand, generates label distributions for biomedical entities, which refine the draft labels from the main classification layer. These label distributions from the BiLSTM architecture provide a more accurate label for the main classification layer by verifying the uncertainty of the draft label (see Fig. 1).

*Uncertainty*. We ask the natural question: *How can we decide what tokens should be refined?* We choose this criterion for the certainty of the draft label on token representations (Gui *et al.* 2021). We calculate the uncertainty *U* of the probability distributions of the MLP layer as follows: where *C* denotes the number of predefined entity types per dataset. We use the entropy of the probability distribution $p_{c}$ to define the uncertainty *U*. For instance, in Fig. 4, we show a situation where the modifier token “colorectal” has a high uncertainty score (0.8) and its draft label needs to be refined. Hence, we present a label refinement process aimed at improving the label distributions of uncertain tokens within biomedical entities.


(7) $$U=H(p_{c})=-\sum_{c=1}^{C}p_{c}\;\;\text{log}\;\;p_{c}.$$

*Label refinement*. We decide to make a consistent label distribution on modifiers (i.e. adjectives or prepositions) used in the entities during training. Specifically, we create an entity-aware mask tensor $\mathrm{MASK}\in R^{B\cdot N\cdot N}$ to indicate the position of the padded indices such that our model does not attend to nonentities, where *B* denotes the batch size. The mask tensor is applied to the BiLSTM architecture $M_{\text{LSTM}}$ to obtain a refined label distribution l, as follows, where *m* denotes a context-dependent representation obtained by BiLSTM architecture, especially for biomedical entities and *l* denotes a refined label distribution to shift the draft label. Then, $M_{\text{LSTM}}$ trains to softly assist the main classification layer in performing precise predictions. Note that we use the term *softly assist* to describe a process that assists the main classification layer in shifting its draft label during training. That is, the trained model $M_{\text{LSTM}}$ enhances the label distribution of an uncertain token and assists in refining the draft label of this uncertain token. Formally, we calculate a label loss $L_{\text{label}}$ to assist predictions as below. where $y_{j}$ and $l_{j}$ denote the ground-truth label and probability of the $M_{\text{LSTM}}$ layer, respectively. To compute the classification loss $L_{\text{class}}$, we add element-wisely to assist predictions as below, where $p_{j}$ denotes the probability of the main classification layer with the assistance of the refinement process. Finally, we determine the criterion using the precomputed uncertainty score *U* in Equation (7). We set an uncertainty threshold Γ to distinguish the tokens that should be refined within entities. For example, in Fig. 4, a token “colorectal” of the entity “colorectal cancer” is first predicted as an Outside tag. During training, the token “colorectal” is converted to a Beginning tag because of the high uncertainty score. We found that Γ=0.3 worked well in practice. See Supplementary Data for an ablation study.


(8) $$m=M_{\text{LSTM}}(\mathrm{MASK}(h))\in R^{N\cdot d}.$$

(9) $$l=MLP(m)\in R^{N\cdot C}.$$

(10) $$L_{\text{label}}=-\frac{1}{N}\sum_{j=1}^{N}y_{j}\cdot \;\text{log}(l_{j})$$

(11) p=p ⊕ l,

(12) $$L_{\text{class}}=-\frac{1}{N}\sum_{j=1}^{N}y_{j}\cdot \;\text{log}(p_{j})$$

*Distillation*. We propose improving the label distributions of biomedical entities by distilling knowledge (Hinton *et al.* 2015) from our decoding layers. We minimize the Kullback-Leibler divergence between the probability distribution from the tag-independent layer (MLP) and the tag-dependent layer (BiLSTM). The distillation loss was computed as follows: where *p* and *l* denote the probability distributions of the MLP and BiLSTM layers, respectively. Note that distillation is computed before Equation (11).


(13) $$L_{\text{distill}}=\frac{KL(p||l)+KL(l||p)}{2},$$

### 3.3 Training objective

**Figure 5:**
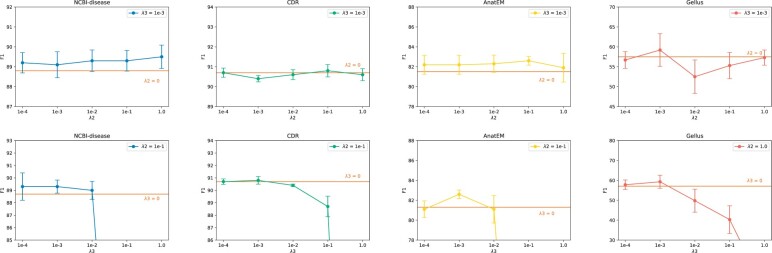
Sensitivity analysis of ConNER components. All the test performances are derived from the best checkpoint of the dev set. We perform five random seeds and compute their mean and standard deviation.

We optimize the three losses altogether to make a consistent label distribution of the entity through label refinement and distilling knowledge while predicting on the tag-independent layer. Our final loss is computed as follows: where $\lambda_{1},\lambda_{2},\lambda_{3}$ scale the importance of each loss term. We observe that $\lambda_{1}=1$, $\lambda_{2}=1e-1$, and $\lambda_{3}=1e-3$ exhibit the best performance in our framework. See Fig. 5 for sensitivity analysis of the other components.


(14) $$L=\lambda_{1}L_{\text{class}}+\lambda_{2}L_{\text{label}}+\lambda_{3}L_{\text{distill}}$$

## 4 Experimental setup

### 4.1 Dataset

We use four biomedical NER benchmarks across four entity types: NCBI-disease (Doğan *et al.* 2014), CDR (Li *et al.* 2016), AnatEM (Pyysalo and Ananiadou 2014), and Gellus (Kaewphan *et al.* 2016), following the standard train/dev/test splits for biomedical NER evaluation. (i) NCBI-disease (Doğan *et al.* 2014) consists of 793 PubMed abstracts with manually annotated disease entities. (ii) CDR (Li *et al.* 2016) contains 1500 PubMed abstracts manually annotated with disease and chemical entities in the same context. (iii) AnatEM (Pyysalo and Ananiadou 2014) consists of 1212 PubMed abstract and full-text extracts annotated with 12 anatomical entity types. (iv) Gellus (Kaewphan *et al.* 2016) consists of annotating cell lines in 1212 documents from PubMed abstracts and PMC full-text extracts. Half of the corpora were drawn from the AnEM corpus (Ohta *et al.* 2012), and the other half were drawn from the BioNLP ST’13 Cancer Genetics (CG) task documents Pyysalo *et al.* (2013). Table 3 shows the dataset statistics.

**Table 3.** Statistics of four biomedical named entity recognition datasets.^a^

Corpora	Entity type	Documents	Sentences	Tokens	Annotations	Unique ann.
NCBI-disease	Disease	793	7142	180 992	6881	2502
CDR	Disease/Chemical	1500	14 503	346 019	12 957/15 837	4477/3765
AnatEM	Anatomic	1212	11 809	298 780	13 692	5042
Gellus	Cell Lines	1212	11 809	312 584	650	243

a The CDR dataset suggests two entity types that share the same text. Annotations (Ann.) and Unique Ann. refer to the entire entity and its unique numbers respectively.

### 4.2 Comparison methods

We evaluate the sentence- and document-level contexts and compare ConNER with several neural network models commonly used in biomedical domains. (i) B-MTM (Crichton *et al.* 2017) developed a multi-task learning model using various biomedical sources annotated with different entity types. (ii) BiLSTM-CRF (Habibi *et al.* 2017) proposed a combination of word embeddings and LSTM with the CRF decoding strategy in the biomedical domain. (iii) BioBERT (Lee *et al.* 2020b) introduced a biomedical-specific language representation model pretrained on large-scale biomedical corpora. (iv) BioLM (Lewis *et al.* 2020) suggested a biomedical-specific language representation model pretrained on biomedical and clinical corpora. (v) CL-KL and CL-L2 (Wang *et al.* 2021) proposed a method that can retrieve and select a semantically relevant context using a search engine to improve contextual representations, with the original sentence as a query. (vi) DocL-NER (Gui *et al.* 2021) proposed a method that leverages a memory network to enhance label consistency at a document level. The difference between DocL-NER and our ConNER is that we use a trainable method to enhance label consistency in label refinement rather than using the memory network. To treat biomedical entities, We train ConNER using BioLM (Lewis *et al.* 2020b) which is commonly used to generate context-dependent representations in the biomedical domain.

### 4.3 Experimental results

Table 4 reports the results of ConNER approach. To prove its effectiveness, we evaluate the model on four biomedical domains in NER tasks and compare it with other methods. Our approach outperformed previous biomedical NER models on three datasets [NCBI-disease (Doğan *et al.* 2014), CDR (Li *et al.* 2016), and AnatEM (Pyysalo and Ananiadou 2014)] and achieve the best results compared to all other baselines. This shows that improving the label consistency of modifier predictions is effective in a document context. The performance gap between BioLM and ConNER on datasets such as AnatEM (74.9 versus 83.5) and Gellus (55.9 versus 63.4) demonstrates that the label refinement based on low label consistency of the dataset bias is effective. In addition, the DocL-NER (Gui *et al.* 2021) performances consistently improved when using token embedding of BioLM in all benchmarks, but our ConNER approach significantly outperforms in AnatEM and Gellus datasets. Although it still lags behind the BiLSTM-CRF model (Habibi *et al.* 2017), the gap has been reduced. We provide further ablation studies of ConNER approach in the following sections.

**Table 4.** Results on biomedical NER benchmarks.^a^

Context	Model	Evaluation (F1)			
		NCBI-disease	CDR	AnatEM	Gellus
Sentence	B-MTM (Crichton *et al.* 2017)^b^	80.4	89.2	82.2	
	BiLSTM-CRF (Habibi *et al.* 2017)^b^	84.6			**75.6**
	BioBERT (Lee *et al.* 2020b)	89.0	89.1	73.9	54.9
	BioLM (Lewis *et al.* 2020)	88.3	89.5	74.9	55.9
	CL-L2 (w/o context) (Wang *et al.* 2021)^b^	89.2	90.7		
	CL-KL (w/o context) (Wang *et al.* 2021)^b^	89.2	90.7		
Document	CL-L2 (w/context) (Wang *et al.* 2021)^b^	89.2	91.0		
	CL-KL (w/context) (Wang *et al.* 2021)^b^	89.0	90.9		
	DocL-NER (Glove) (Gui *et al.* 2021)	88.8	90.5	70.7	52.5
	DocL-NER (BioLM) (Gui *et al.* 2021)	89.2	91.1	76.2	55.7
	ConNER (Ours)	**89.9**	**91.3**	**83.5**	63.4

a F1 score is reported. All the test performances are derived from the best checkpoint of the dev set. The best score is displayed in bold and the second-best score is underlined.

b Numbers are estimated from the figures in the original papers.

## 5 Analysis

### 5.1 Sensitivty analysis of $\lambda_{2}$ and $\lambda_{3}$

To understand the impact of two objectives $L_{\text{label}}$ and $L_{\text{distill}}$, we conduct a sensitivity analysis of the ConNER components. The results of this analysis are shown in Fig. 5, which displays the performance changes with different values of coefficients $\lambda_{2}$ and $\lambda_{3}$. The orange line signifies ablation results without corresponding loss functions ($\lambda_{2}=0$ or $\lambda_{3}=0$). The best and most stable performance is achieved when $\lambda_{2}=1e-1$ and $\lambda_{3}=1e-3$. However, if the value of $\lambda_{3}$ exceeds 1e–2, the performance drops significantly. This suggests that overly constraining $\lambda_{3}$ can negatively affect the prediction of the biomedical entity label distributions.

### 5.2 Consistency enhancement of shorter entities

**Figure 6:**
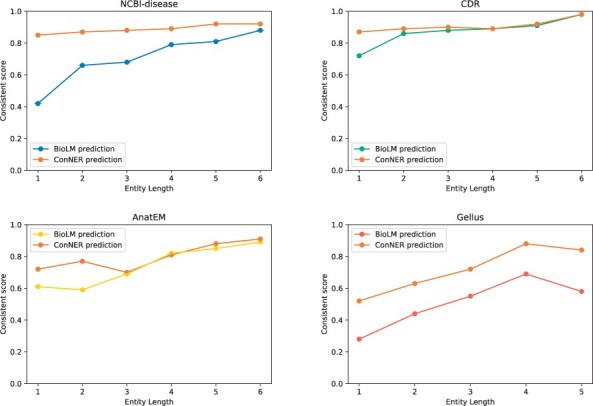
Consistent enhancement of ConNER predictions per entity length. Compared to BioLM predictions, a higher gap of consistency score shows when the entity length is 1.

In Fig. 2, we observe that entities with shorter lengths ($\phi_{\text{eLen}}<5$) have a low label consistency, which mostly contains modifiers. Our ConNER approach aims to tackle these shorter entities. In Fig. 6, we compare the predictions of BioLM and ConNER to evaluate the consistency of shorter entities. Compared to BioLM, our ConNER approach demonstrates a higher consistency score, particulary when the entity length is only 1 ($\phi_{\text{eLen}}=1$). The plot shows a consistent improvement in all datasets and after the length surpasses 6, the consistency score of both models is nearly equal. These plots indicate that our ConNER approach effectively addresses the issue of short entities.

### 5.3 Ablation studies

We also see if our ConNER approach can improve the label consistency of entities containing modifier tokens. Table 5 shows the cases of inconsistent predictions in document NER models. We analyze the modifier tokens used as both entity and nonentity tokens depending on the context. For example, in the NCBI-disease dataset (Doğan *et al.* 2014), we observe that BioLM trained on document context did not predict consistently on these modifier tokens. In addition, DocL-NER method shows consistent predictions in these tokens due to the label refinement of the memory network. Owing to the improvement of label consistency on the uncertain tokens within entities, the ConNER approach achieves consistent predictions on these tokens.

**Table 5.** Case studies of inconsistent predictions in document NER models.^a^

Data	Criteria	Modifier token examples					
		Primary (%)	Genetic (%)	Hereditary (%)	Inherited (%)	Congenital (%)	Abnormal (%)
Dev	BioLM	5	52	50	71	78	63
	DocL-NER	78	66	93	88	89	65
	ConNER	92	78	92	91	95	79

a The below case was taken from the NCBI-disease dev dataset Doğan *et al.* (2014). The predictions of BioLM (Lewis *et al.* 2020), DocL-NER (Gui *et al.* 2021), and our ConNER approach are compared. Our ConNER approach achieves consistent predictions on the modifier examples.

### 5.4 Qualitative analysis

Table 6 shows our ConNER prediction on the AnatEM dataset. We looked into examples that highlight the strengths and weaknesses of our approach and found three different cases: (1) Our model predicts the text “rectal carcinoma” consistently as an entity., while another model without label refinement and distillation process predicts the word “rectal” as an entity in its first appearance and as a nonentity in its second appearance. (2) A surprising case is the word “surgical.” In the training dataset, it had a consistency score of 0.923 in the entity dictionary, meaning it was mostly used as an entity, accounting for 92.3% of the total. Our ConNER approach performs well in predicting the “surgical” as an entity. (3) However, our approach has limitations in generalizing on out-of-density tokens like “mesorectum” or “margin” which never occurred in the training dataset. Our approach cannot predict these tokens as biomedical entities, which is a common situation in real-world scenarios. In addition, when comparing the predictions for “peritumoral lymphocytic” and “lymph nodes,” we see that the token “lymph” was predicted inconsistently despite being part of the same entity. We discover that this was due to the low degree of label consistency in the given paragraph or the entire dataset (0.62 consistency score). In future work, we will look into ways to make our approach more generalizable for out-of-density tokens and more consistent for inconsistent tokens.

**Table 6.** A sample prediction of ConNER on the AnatEM and CDR datasets.^a^

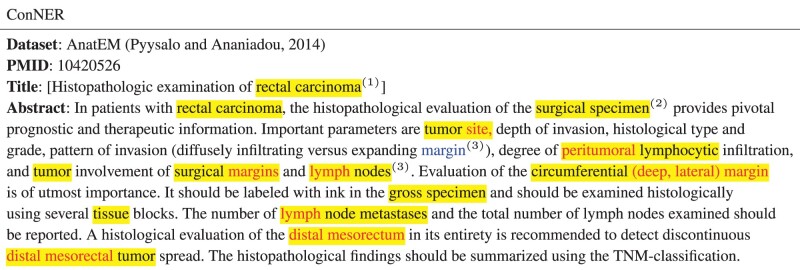

a Yellow highlight refers to the golden answer, and red signifies to the false negative prediction, and blue signifies to the false positive prediction.

## 6 Conclusion

In this article, we introduce the ConNER approach, which improves label consistency in biomedical named entity recognition (NER) models in document contexts. Our approach includes a label refinement process to encourage consistent label prediction of modifier tokens within entities. The ConNER approach outperforms existing biomedical NER methods in three biomedical datasets and provides insight into the relationship between label consistency attributes and performances. Additionally, our approach performs well on low-resource datasets, demonstrating its potential for biomedical NER applications. In future work, we aim to address the challenge of out-of-density tokens, which are not present in the training data, by using not only annotated paragraphs but also relevant contexts retrieved based on these tokens.

## Supplementary Material

btad361_Supplementary_Data
