# Supplementary material for: Consistency enhancement of model prediction on document-level named entity recognition
Source: Bioinformatics. 2023 Jun 1;39(6):btad361. doi: 10.1093/bioinformatics/btad361 (PMC10272703; doi:10.1093/bioinformatics/btad361)
Supplement: btad361_Supplementary_Data [file btad361_supplementary_data.pdf]

Supplementary data for

Consistency Enhancement of Model Prediction on Document-level Named Entity Recognition

Minbyul Jeong [1] and Jaewoo Kang [1,2,3,*]

[1] Department of Computer Science and Engineering, Korea University, Seoul, 02841, Republic of Korea
[2] Interdisciplinary Graduate Program in Bioinformatics, Korea University, Seoul, Republic of Korea
[3] AIGEN Sciences, Seoul, 04778, Republic of Korea

* To whom correspondence should be addressed.

Availability and implementation: Our code and resources are available at https://github.com/dmis-lab/ConNER

Contact: kangj@korea.ac.kr

A Implementation Details

Table S1. Hyperparameters of the four biomedical named entity recognition settings. For common hyperparameter settings, we focus on searching batch size, learning rate, and training epoch for learning schemes. We also focus on searching for the optimal hyperparameters of the uncertainty threshold Γ.

Context	Corpora	Pretrained LM	Batch Size	Learning Rate	Training Epoch	Sequence Length	Threshold Γ	λ_2	λ_3
Document	NCBI-disease	BioLM	6	3e-5	50	512	0.3	1e-1	1e-3
	CDR	BioLM	6	3e-5	30	512	0.3	1e-1	1e-3
	AnatEM	BioLM	6	5e-5	30	512	0.3	1e-1	1e-3
	Gellus	BioLM	6	3e-5	40	512	0.3	1.0	1e-3

We train ConNER using BioLM (Lewis *et al.*, 2020a) to treat biomedical entity types. This pre-trained language model is commonly used as a backbone model in the biomedical domain, and we adopt this model to generate contextualized representations. We set 128 tokens as the maximum sequence length for the sentence-level context and 512 tokens for the document-level context, and tokens with more than the maximum sequence length were truncated. The batch size was set to 32 for the sentence level and 6 for the document level. We select a learning rate in the range {3e-5, 5e-5}. We search for a training epoch in the range {30, 40, 50}. We suggest our total hyperparameter settings in Table 1. We train our model with a single NVIDIA Titan RTX (24GB) GPU for fine-tuning, and the training time took less than 2 hours.

Table S2. Breakdown of NCBI-disease dev set error cases that BioLM trained on document context. The highlight signifies a golden answer.

**False Negative (81%)**
**Misprediction of entity boundary with modifiers (49%)**
Text: ...heterogeneity in hereditary breast cancer : role of BRCA1 and BRCA2 ...<br>Prediction: breast cancer
**Misprediction of entity boundary without modifiers (16%)**
Text: Adult onset globoid cell leukodystrophy (Krabbe disease) : analysis of ...<br>Prediction: globoid cell leukodystrophy, Krabbe disease
**Cannot predict as an entity (13%)**
Text: tumorigenesis, but not in resistance to acute radiation toxicity .<br>Prediction:
**Blank between predictions (3%)**
Text: Molecular basis for Duarte and Los Angeles variant galactosemia .<br>Prediction: Duarte and Los Angeles, galactosemia
**False Positive (19%)**
**Misprediction of entity boundary with modifiers (15%)**
Text: a frequent cause of idiopathic Addison ' s disease in young adult male patients .<br>Prediction: idiopathic Addison ' s disease
**Predict as an entity which is not an entity (4%)**
Text: the WASP gene defects found in patients with severe disease .<br>Prediction: WASP gene defects

B Breakdown of Error cases

In Table 2, we break down the error cases of BioLM trained on document context in the NCBI-disease validation dataset (Doğan *et al.*, 2014). We derive the 100 random cases to classify the predictions as false negative (81%) and false positive (19%). We focus on the largest proportion of errors in our case, which is the misprediction of entity boundaries. Many of these cases included modifiers such as adjectives and prepositions (false negative 49% and false positive 15%). Upon examining the attribute functions in our training dataset, we find that the consistency score of entities containing modifiers was generally low. To address this, we propose the ConNER approach to make more consistent predictions.

C Consistency Enhancement on predictions

Table 3 shows the sample predictions of BioLM (left column) and ConNER(right column) on NCBI-disease dataset. Each row shows a portion of an entire paragraph, and it demonstrates the improvement of inconsistent predictions to consistent ones. Most of the entities are shown to have modifiers in short-length entities and a considerable number of predictions are not consistent. We can see that through the

Table S3. Sample predictions of BioLM and ConNER on the NCBI-disease dataset. yellow highlight refers to the golden answer, and red signifies to the false negative prediction.

BioLM Predictions	ConNER Predictions
Mutations in BRCA1 account for approximately 45% of familial breast cancer and 90% of inherited breast / ovarian cancer , whereas mutations in BRCA2 account for a comparable percentage of inherited breast cancer cases .	Mutations in BRCA1 account for approximately 45% of familial breast cancer and 90% of inherited breast / ovarian cancer , whereas mutations in BRCA2 account for a comparable percentage of inherited breast cancer cases .
apoptosis of human prostate carcinoma mediated by a genetic locus within human chromosome 10pter - q11. Prostate cancer is the second leading cause of male cancer deaths in the United States . Prostate secretory epithelial cells and androgen - dependent prostate carcinomas undergo apoptosis in response to androgen deprivation and , furthermore , most prostate carcinomas become androgen ...	apoptosis of human prostate carcinoma mediated by a genetic locus within human chromosome 10pter - q11. Prostate cancer is the second leading cause of male cancer deaths in the United States . Prostate secretory epithelial cells and androgen - dependent prostate carcinomas undergo apoptosis in response to androgen deprivation and , furthermore , most prostate carcinomas become androgen ...
The spectrum of RB1 germ - line mutations in hereditary retinoblastoma . searched for germ - line RB1 mutations in 119 patients with hereditary retinoblastoma .	The spectrum of RB1 germ - line mutations in hereditary retinoblastoma . searched for germ - line RB1 mutations in 119 patients with hereditary retinoblastoma .
Increased coronary heart disease in Japanes - American men with mutation in the cholesteryl ester ... strongly genetically determined and show a general inverse relationship with coronary heart disease (CHD) .	Increased coronary heart disease in Japanes - American men with mutation in the cholesteryl ester ... strongly genetically determined and show a general inverse relationship with coronary heart disease (CHD) .

proposed label refinement process, we can get more consistent predictions in a considerable number of contexts.

D Ablation Studies

D.1 Performance of removing each loss term

Table 4 shows our ablation result on four biomedical NER benchmarks. We evaluate our approach ConNER by removing its components: 1) distillation (—$L_{distill}$) and 2) the label-refinement process (—L_{label}). The experiments show that ConNER is effective for all four benchmarks. Specifically, we observe that the AnatEM and Gellus datasets show significant improvement, demonstrating that our approach ConNER is effective for datasets with low label consistency on the entities. We also observe that adding each component consistently improves the recall metrics. These observations correspond to an advantage of ConNER approach, whereby it decides which token should be refined by relying on the uncertainty threshold Γ.

Table S4. An ablation study of ConNER components. We perform three different experiments: removing distillation (— $\{L_{distill}\}$), label refinement (— $\{L_{label}\}$), and both of these process (— $\{L_{distill}, L_{label}\}$). We used our best hyperparameter setting on the BioLM (Lewis et al., 2020b) model equally. The best scores are displayed in bold.

Model Description	Evaluation (P/R/F1)			
	NCBI-disease	CDR	AnatEM	Gellus
ConNER	**88.8 / 91.1 / 89.9**	**89.9 / 92.7 / 91.3**	**83.2 / 83.7 / 83.5**	**62.6 / 64.2 / 63.4**
ConNER — $\{L_{distill}\}$	87.5 / 89.9 / 88.7	89.2 / 92.3 / 90.7	81.6 / 81.1 / 81.3	54.3 / 60.3 / 57.1
ConNER — $\{L_{label}\}$	87.4 / 90.4 / 88.8	89.4 / 92.1 / 90.7	81.0 / 81.9 / 81.5	54.2 / 61.2 / 57.5
ConNER — $\{L_{distill}, L_{label}\}$	87.4 / 89.7 / 88.5	89.8 / 90.7 / 90.2	80.3 / 80.4 / 80.3	54.8 / 59.1 / 56.9

D.2 Impact of threshold Γ

We provide an interpretation of the threshold Γ (see Figure 1). We observe that $\Gamma = 0.3$ works well in our four benchmarks, except for the Gellus dataset. When Γ is higher than 0.6 in the NCBI-disease dataset, the F1 score yields the same results. We analyze the situation in which a biomedical pre-trained language model has already demonstrated stable performance in predicting biomedical entities. We find that ConNER approach does not have to interfere with the predictions of the main classification layer. In contrast, in the Gellus dataset, $\Gamma = 0.8$ shows the best performance with high precision and recall metrics. The results were found to be steady when we compute five times using different seeds. However, we suggest using $\Gamma = 0.3$ to achieve a stable performance on all benchmarks.

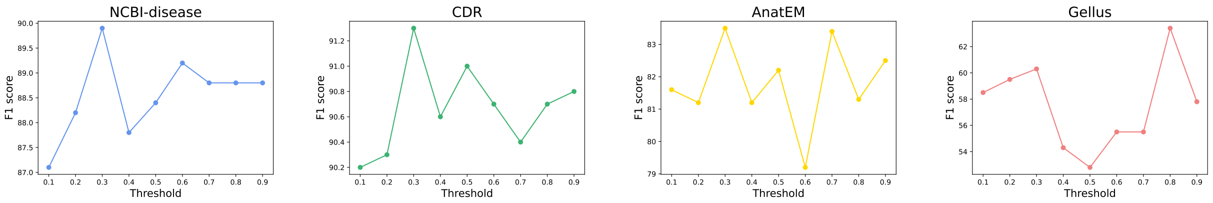

Fig. S1: An analysis of uncertainty threshold Γ. The x-axis refers to the threshold Γ, and the y-axis denotes the F1 score. The overall results show that $\Gamma = 0.3$ results in stable performance on the four biomedical benchmarks. All the test performances are derived from the best checkpoint of the dev set.

Funding

This work was supported in part by the National Research Foundation of Korea (NRF-2020R1A2C3010638), the Ministry of Health & Welfare, Republic of Korea (HR20C0021), and the MSIT(Ministry of Science and ICT), Korea, under the ICT Creative Consilience program(IITP-2022-2020-0-01819) supervised by the IITP(Institute for Information & communications Technology Planning & Evaluation).

References

Doğan, R. I. *et al.* (2014). Ncbi disease corpus: a resource for disease name recognition and concept normalization. *Journal of biomedical informatics*.

Lewis, P. *et al.* (2020a). Pretrained language models for biomedical and clinical tasks: Understanding and extending the state-of-the-art. In *Proceedings of the 3rd Clinical Natural Language Processing Workshop*.

Lewis, P. *et al.* (2020b). Pretrained language models for biomedical and clinical tasks: Understanding and extending the state-of-the-art. In *Proceedings of the 3rd Clinical Natural Language Processing Workshop*.
